# An Extraordinary Case of Small-Pox

**Published:** 1801-05

**Authors:** Thomas Purton

**Affiliations:** Member of the Royal College of Surgeons in London.


### *An extraordinary Case of Small-Pox, by* THOMAS PURTON, *Member of the Royal College of Surgeons in London.*

To the EDITORS of the MEDICAL and PHYSICAL JOURNAL.

Gentlemen,

IT has been so generally considered as a fact, that a person once infected with the small-pox is safe from having it a second time, that I feel a considerable degree of diffidence in sending you the following statement of a case, by which it is indisputably proved, that it is *possible* for the variolous infection to take place a *second time* in the *same person*, and that *even* in the *natural way*.

My partner, Mr. Bloxam, a gentleman of sound judgment, who has been established forty years in a very extensive business, and who visited the patient with me through every stage of the disease, has not a doubt of the nature of the case; nor should I have troubled you on the present occasion, if my mind had not been thoroughly convinced of its accuracy.

I have been told by an elderly lady of respectability, that she knew a person who certainly had had the small-pox twice; and her statement was so very accurate and circumstantial, that it was sufficient to stagger the firmest sceptic; but I confess that my opinion remained unchanged until the following case came under *my own* observation.

The circumstance of a person having the small-pox a second time, *from its rarity*, must be considered as a singular phœnomenon in pathology; therefore, I cannot perceive in what way (even if it was generally known) it can injure the cause of variolous inoculation; but it decisively proves that the small-pox may in a few rare cases be received twice by the same person, and therefore it ought to be known; and this case deserves more particular attention, because the subject of it was not inoculated for the small-pox, but received the disease in the casual way on both occasions. In the first attack, her mother caught the disease from her, and narrowly escaped with her life. The last time I saw her myself; and any person who has doubts, may be satisfied by inoculating with some of the matter, which I have preserved.

This case is farther entitled to notice, because no greater security against the small-pox can be expected from the Vaccine Inoculation than from the infection of the natural small-pox; therefore the preference of the cow-pox would not be invalidated by the occurrence of an occafional cafe, in which the fmall-pox might be caught after inoculation for the cow-pock, if fuch an inftance fhould arife.

The fubject of the prefent cafe is Maria Hunt, of Exhall, near this town, *aged twenty-two years*; fhe caught the fmall-pox when fhe was *five months old*, and had them very violent, as the marks at this time fufficiently teftify.—Her mother caught the difeafe of her, and had it to fo alarming a degree, that the apothecary who attended, had fcarcely a hope of her recovery.—The mother is ftill living, from whom I received this account, which was alfo corroborated by the teftimony of many refpectable neighbours.

On the 11th of March laft, the whole village of Exhall was inoculated with variolous matter, and this young woman was appointed one of the nurfes.—On the 3d of April, fhe was attacked with fevere rigors, pain in the head and back, ficknefs, and other ufual fymptoms of an approaching fever.—The two following days fhe was extremely ill, the fever increafed to an alarming height, attended with delirium, through moft part of the night previous to the appearance of the eruption, which fhewed itfelf on the 6th of April, early in the morning. The eruption continued to increafe for feveral days; her throat became fore, with a fenfe of fullnefs, from the number of puftules covering the fauces; and they continued filling till they were completely maturated.—About the 9th day from the appearance of the eruption, thofe upon her face began to turn, and in a few days after, thofe upon the reft of her body.

I never thought of taking any matter till the thinner contents of the puftules were nearly abforbed; but, however, I have obtained a very fufficient quantity, part of which I will fend to you, or any gentleman who wifhes for farther confirmation, to make trial of it.

The above is a juft detail of this extraordinary cafe, which I leave to medical men to make their own comments upon.

I am, &c.

*Alcester, April* 17, 1801. T. PURTON.

